# Sugarcane Extract (Polygain™) Supplementation Reduces Enteric Methane Emission in Dairy Calves

**DOI:** 10.3390/ani15060781

**Published:** 2025-03-10

**Authors:** Richard Osei-Amponsah, Pragna Prathap, Frank R. Dunshea, Richard Eckard, Matthew Flavel, Muhammed Elayadeth-Meethal, Surinder S. Chauhan

**Affiliations:** 1 School of Agriculture, Food and Ecosystem Sciences, The University of Melbourne, Parkville, Melbourne, VIC 3010, Australia; rosei-amponsah@ug.edu.gh (R.O.-A.); pragnaprathap@gmail.com (P.P.); fdunshea@unimelb.edu.au (F.R.D.); rjeckard@unimelb.edu.au (R.E.); muhammed.elayadethmeethal@unimelb.edu.au (M.E.-M.); 2 Department of Animal Science, School of Agriculture, University of Ghana, Accra P.O. Box LG 25, Ghana; 3 Meat and Wool Services, Agriculture Victoria, Department of Energy, Environment and Climate Action, Ellinbank, VIC 3821, Australia; 4 Faculty of Biological Sciences, The University of Leeds, Leeds LS2 9JT, UK; 5 The Product Makers (Australia) Pty Ltd., 50-60 Popes Rd, Keysborough, Melbourne, VIC 3173, Australia; mflavel@tpm.com.au

**Keywords:** ruminant nutrition, dairy cattle, feed additives, polyphenols, climate-smart livestock

## Abstract

Polygain™ (PG), a polyphenolic extract from sugarcane, has recently been identified as a potential additive to reduce methane (CH_4_) emissions in livestock. This experiment examined the effects of PG on the enteric CH_4_ emission from Holstein Friesian weaned calves. Calves were allocated to annual pasture grazing and received supplementary pellets (200 g/calf/day; Barastoc calf-rearer cubes—Ridley Corporation). The experimental design followed was a completely randomized design (CRD), comprising 24 female calves (4–5 months old) allocated to two equal groups; control (standard pellets) vs. treatment (pellets formulated by adding PG to control pellets to deliver 10 g PG/calf/day). Experimental diets were fed for three months between August and November 2023, including a two-week adaptation period. Calves were weighed at the start and at the end of the study. A GreenFeed (C-Lock Pvt Ltd.) emission monitoring unit (GEM) was used to measure GHG emissions from the experimental calves in their groups in a 2-day rotational cycle. During a visit to the GEM, the calves were encouraged to enter an enclosed area or individual feeding stall where enteric CH_4_, CO_2_, O_2_, H_2_, and H_2_S measurements were taken. The results indicated a significant effect of PG supplementation on enteric methane emission in calves, with a lower production of CH_4_ in calves supplemented with PG (26.66 ± 2.06 g/day) as compared to the control group (35.28 ± 1.39 g/day, *p* < 0.001). The CO_2_/O_2_ ratio in the treatment (235 ± 14) and control groups (183 ± 9.6) differed significantly (*p* < 0.001). Overall, PG supplementation (10 g/calf/day) reduced their average methane emission per day and did not adversely affect the growth and development of experimental calves, confirming its useful anti-methanogenic potential.

## Simple Summary

Greenhouse gases (GHGs) are the primary drivers of global warming and climate change, with carbon dioxide (CO_2_) and methane (CH_4_) being the two most significant GHGs. In ruminants, enteric fermentation is responsible for 80–90% of GHG emissions from livestock. Consequently, various animal research efforts seek to reduce the quantity of methane produced by ruminants through nutritional strategies and potential genetic selection interventions. In this study, we investigated the effects of Polygain™ (PG), a polyphenolic extract from sugarcane, on the enteric CH_4_ emission from Holstein Friesian weaned calves kept at the University of Melbourne Dookie Dairy Farm. Our findings indicate that PG supplementation reduced their average methane emission per day and did not adversely affect the growth and development of experimental calves. Our results confirm the anti-methanogenic potential of PG, which provides another potential dietary additive that can help the livestock industry reduce methane emissions and promote sustainable dairy cattle production in the face of changing climates. By introducing methane-mitigating feed additives, such as PG to calves, the composition and activity of the rumen microbial community may be influenced, resulting in reduced methane production. This early-life intervention may be a potential strategy for long-term methane mitigation as calves mature into adult ruminants.

## 1. Introduction

Ruminant livestock production is dependent on the anaerobic microbial ecosystem (including bacteria, archaea, protozoa, and fungi) to ferment and transform human indigestible forages into high-grade dairy and meat products for human consumption [1]. This enteric fermentation process is responsible for 80–90% of GHG emissions from livestock. Greenhouse gases (GHGs) are the primary drivers of global warming and climate change. The two significant GHGs are CO_2_ and methane (CH_4_) [2]. In general, ruminant production systems contribute up to 18% of total global greenhouse gas (GHG) emissions, primarily through enteric methane (CH_4_), which is the most common source of GHGs. Enteric fermentation accounts for over 90% of CH_4_ emissions from livestock and 40% of agricultural GHG emissions [3]. Ruminant livestock such as cattle, sheep, and goats have diverse microbial communities in their stomach that use anaerobic fermentation to digest feed, releasing CH_4_ as a byproduct. Methane production may be enteric or through manure. However, enteric emission is the major contributor, accounting for 90% of total methane produced by cattle [4]. Enteric methane contributes 30% of the CH_4_ released into the atmosphere daily, more than any other single source [5]. Strategies to reduce ruminant CH_4_ emissions would not only decrease GHG emissions but will also increase the energy available for growth and production [5]. Feed additives, such as algae, saponins, tannins, and essential oils, have emerged as promising strategies to mitigate CH_4_ emissions from ruminant livestock [6,7,8]. While it is true that plant extracts can assist in reducing methane emissions, it is crucial to source and produce plants in a sustainable manner [3]. These plant-based supplements can modify the microbiota and reduce CH_4_ production in ruminants [6,7]. They offer environmental benefits and economic advantages, such as improved feed efficiency and growth rates [8,9]. Polygain™ (PG) (The Product Makers Australia Pty Ltd. Melbourne, VIC 3173, Australia), a sugarcane extract feed additive rich in polyphenols, has anti-methanogenic properties [10,11]. Introducing CH_4_-mitigating feed additives to calves during early rumen development is crucial, as this period involves significant microbial colonization and rumen maturation [12,13]. Early supplementation can influence the rumen microbial community, leading to long-term CH_4_ reduction and improved nutrient utilization and growth performance [14]. Low methane production in calves significantly enhances growth by improving feed efficiency and energy utilization [15]. Methane (CH_4_) is a byproduct of ruminal fermentation, representing a substantial energy loss—up to 8–12% of gross energy intake [16,17]. Lower methane production also correlates with increased propionate levels, a volatile fatty acid that is efficiently converted into glucose via gluconeogenesis in the liver [18]. This process enhances energy availability for tissue growth and milk production [19]. Furthermore, early exposure to these additives supports the development of efficient digestion patterns, optimizing nutrient utilization and promoting overall growth performance [20]. Thus, timely implementation of methane-mitigating feed additives during early rumen development may offer substantial benefits for environmental sustainability and long-term productivity [21].

Although Polygain™ has shown effectiveness in reducing CH_4_ emissions in sheep, its impact on calf growth and CH_4_ emissions requires further investigation. On-farm research and climate-smart practice validation are needed to implement these strategies effectively. While some studies have validated feed interventions for CH_4_ mitigation in beef cattle [22], more research is needed on other feed additives. In our previous study on sheep [23], Polygain™ reduced enteric CH_4_ emissions by 49.3% and 33.3% at 0.25 PG and 1 PG (g/kg DMI), respectively, without adverse effects on growth rates and meat quality. Anti-methanogenic feed supplements can be chemicals like nitro compounds (3-Nitrooxypropanol (3-NOP), bromoform, or plant extracts like polyphenols [8]. The nitro compound 3-NOP primarily reduces methane production by directly inhibiting methyl–coenzyme M reductase (MCR), which is the enzyme that catalyses the methane-forming step of methanogenesis in methanogenic archaea in the rumen [24,25]. However, plant metabolites such as polyphenols lower enteric methane production by 8–50% by modifying rumen fermentation towards propiogenesis, and altering the activity, abundance, and diversity of microbes in the rumen [26]. Polyphenols often depress the activity of gram-positive fibrolytic bacteria and ciliate protozoa, resulting in a reduction in volatile fatty acid production (mostly acetate) [27]. In addition to lowering methane production, plant extracts have positive health effects such as antioxidant activity [28]. Hence, compared to 3-NOP or bromoform, plant extracts are safer and more effective when used on calves [27,29].

The objective of the present study, therefore, was to evaluate the potential benefits of feeding PG to weaned calves, including growth improvements and CH_4_ emission reductions. We hypothesized that Polygain™ supplementation would reduce enteric CH_4_ emissions and enhance growth rates in dairy calves.

## 2. Materials and Methods

### 2.1. Experimental Location and Design

This study was conducted at the University of Melbourne Dookie Dairy Farm, with approval from the University of Melbourne Animal Ethics Committee (AEC ID: 2023-27060-43375-3). The Dookie Dairy Farm is in the Southern Hemisphere, in the state of Victoria, Australia on latitude 36.4° S and longitude 145.7 °E (940 Dookie-Nalinga Road, Dookie College, VIC 3647, Australia). The dairy farm has a 43-hectare irrigated pasture with annual average rainfall of 540 mm. The area surrounding the dairy farm is level, with either short grass or bare soils. The mean daily minimum and maximum air temperatures are 10 and 21 °C, respectively.

Calves were housed in an open paddock, grazed on rye-grass pasture (nutritional composition: dry matter—93.5%, crude protein—18.3%, acid detergent fibre—25.7%, neutral detergent fibre—48.1%, digestibility (DMD)—71.3%, metabolizable energy (calculated)—10.6 MJ/kg of DM, water-soluble carbohydrates—6.8% of DM, fat—5.2% of DM, and ash—13.2% of DM), and received supplementary calf-rearer/heifer-developer pellets as per standard husbandry practices. Polygain™ (PG) (The Product Makers Australia, Keysborough, Australia), a polyphenolic extract from Australian-grown sugarcane known for its CH_4_ mitigation potential, was used as a feed additive. Calves were allocated to annual pasture grazing and received supplementary calf-rearer pellets (200 g/calf/day; Barastoc calf-rearer cubes—Ridley Corporation) according to standard farm practices. The experimental design was a completely randomized design (CRD) with 24 female calves (4–5 months old) divided into two equal groups: control (standard pellets) and treatment (pellets with 10 g Polygain™/calf/day). The experimental diets were fed for three months (between August and November 2023), including a two-week adaptation period and weekends. However, in line with animal ethics, animals were rested from the GreenFeed measurements during weekends. During the adaptation period, calves were gradually introduced to the treatment pellets, and their feed consumption was closely monitored. Calves were weighed at the start and end of the study (60-day intervals).

### 2.2. Data Collection

The body weight (BW) of the calves was measured using a weighing scale (Gallagher TSi 2 Livestock Manager, Shepparton, Australia) at the start and end of the study and the average daily gain (kg/d) was calculated [13]. Various methods have been developed to measure CH_4_ emissions from animals, including direct approaches like respiration chambers, the GreenFeed system, sniffer techniques, and open-circuit respiration systems, as well as indirect methods using proxies and emission models. Enteric CH_4_ emission can be reliably measured by the GreenFeed monitoring unit [30] and was used in this study after calibration and standardised training of the research team by the manufacturer, C-Lock Inc. Raw sensor voltage readings were converted to ppm using a standard two-point calibration with CH_4_ (508 ppm) and CO_2_ (4982 ppm). Every month, a CO_2_ calibration was performed to compare the total amount of CO_2_ emitted into the GEM with the amount of CO_2_ that the GEM measured [22].

**Figure 1:**
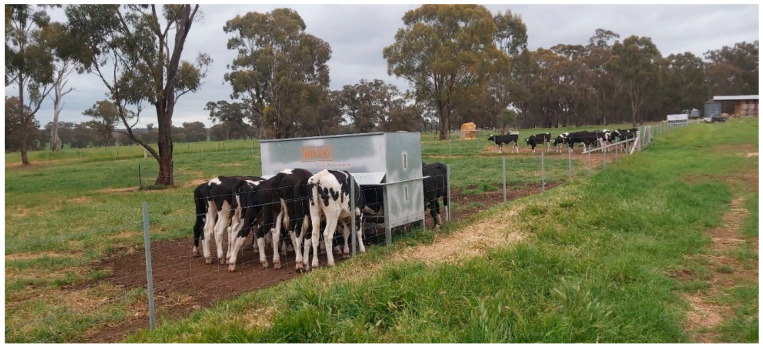
Experimental calves of the treatment group feeding on assigned pellets from a feeder.

The GreenFeed system, created by C-Lock Inc., is an automated head-chamber system that samples CH_4_ emissions and gaseous exchange in ruminants. It includes a head-chamber system coupled with a mobile feeding station [31]. The GreenFeed program regulates the timing and quantity of feed availability for each calf, making sure that the measurements are distributed equally during a 24 h feeding cycle and that data are transferred to a cloud-based analytic system [32]. In this study, the GreenFeed emission monitoring unit (GEM) [22] measured GHG emissions from the experimental calves in their groups in a 2-day rotational cycle. On each of these two days, the calves voluntarily visited the GreenFeed and gas measurements were taken on each visit (Figure 1). The visit period was approximately 3 min for every single visit, but an animal could visit multiple times within 2 days. Overall, the total number of days for GHG measurements was 60 days.

The GEM estimates average daily GHGs, including enteric CH_4_ production (DMP), in a non-invasive manner, providing data without harming or distressing the animals. Each calf was identified using RFID ear tags to accurately track CH_4_ emissions throughout the trial. A bait attractant (pelleted feed) was used to encourage calves to visit the GEM frequently. Within the station, calves could access a feed trough equipped with sensors that analyse their breath samples for CH_4_ emissions. Methane measurements were taken to assess the impact of PG supplementation on CH_4_ emissions.

**Figure 2:**
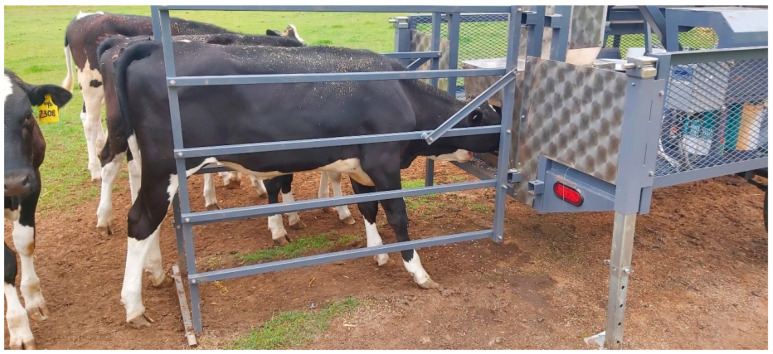
One of the experimental calves during a visit to GreenFeed.

Grazing calves were supplemented with calf-rearer pellets (10 g/calf/day), and additional bait was fed in GreenFeed units used for emission measurements [33]. During visits to the GEM, calves entered an enclosed area or individual feeding stall (Figure 2), where measurements of CH_4_, CO_2_, O_2_, H_2_, and H_2_S were taken.

### 2.3. Data Analysis

Preliminary analyses indicated that body weight and GHG measurements were normally distributed. All data were analysed using the t-test procedure (PROC TTEST) of SAS (version 9.4, SAS Institute Inc., Cary, NC, USA). The statistical model included treatments as fixed effects with calves as random effects:

$$y_{ij}=\mu +b_{i}{+e}_{ij}$$

where $y_{ij}$ is the performance parameter (body weight or GHG emission of calf), μ is the population mean, $b_{i}$ is the treatment group (effect of Polygain™), and $e_{ij}$ is the residual or the random error term. The data for all the studied variables for each calf were averaged across days and were used in the statistical analysis. Means were separated by pairwise t-test (PROC T TEST). Statistical differences were considered significant at *p* ≤ 0.05. Data in tables are presented as least squares means [29]. Pearson correlation coefficients between the emitted GHGs and their ratios were estimated using the PROC CORR procedure of SAS (version 9.4, SAS Institute Inc., Cary, NC, USA). The graphical representations were created using R (version 4.4.2, R Core Team) [34].

## 3. Results

### 3.1. Variation in Calf Body Weight by Group

The two experimental calf groups were similar (*p* > 0.05) in average body weight at the beginning and end of the experiment (Table 1). Average daily gain was also not significantly different (*p* = 0.08) across the groups.

### 3.2. Variation in GHG Emissions by Experimental Group

**Figure 3:**
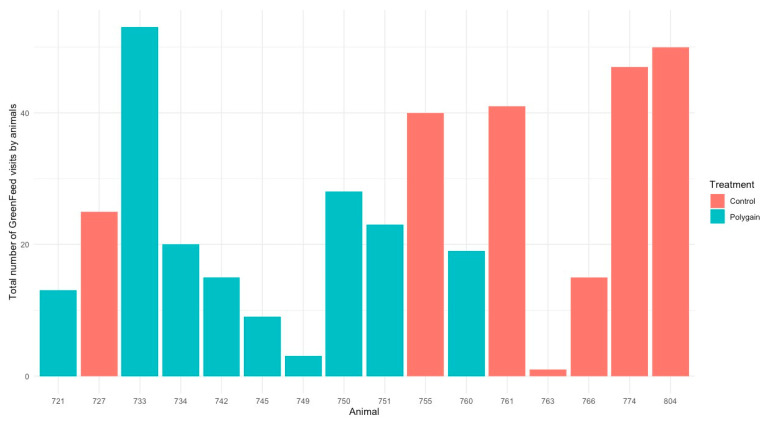
Total number of visits of animals in GreenFeed during the experiment.

**Figure 4:**
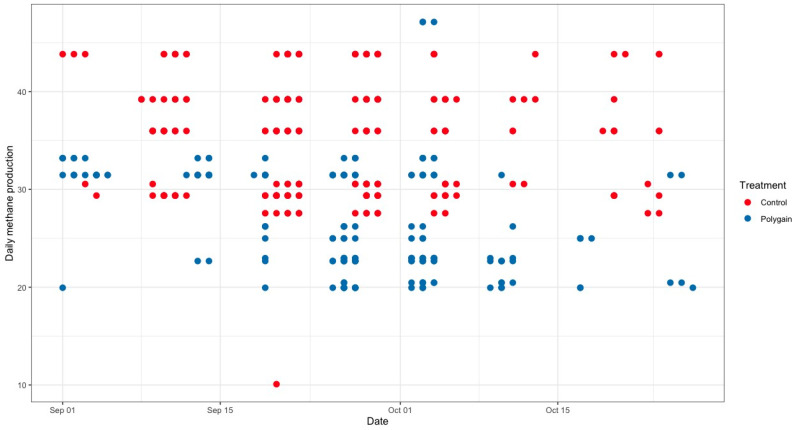
Average daily methane emission (g/calf/day) of control and Polygain™ supplemented calves during the study period.

**Figure 5:**
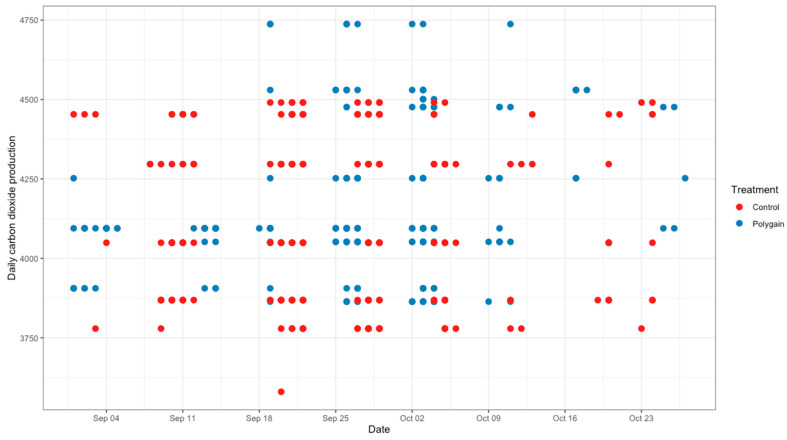
Average daily carbon dioxide emission (g/calf/day) of control and Polygain™ supplemented calves during the study period.

**Figure 6:**
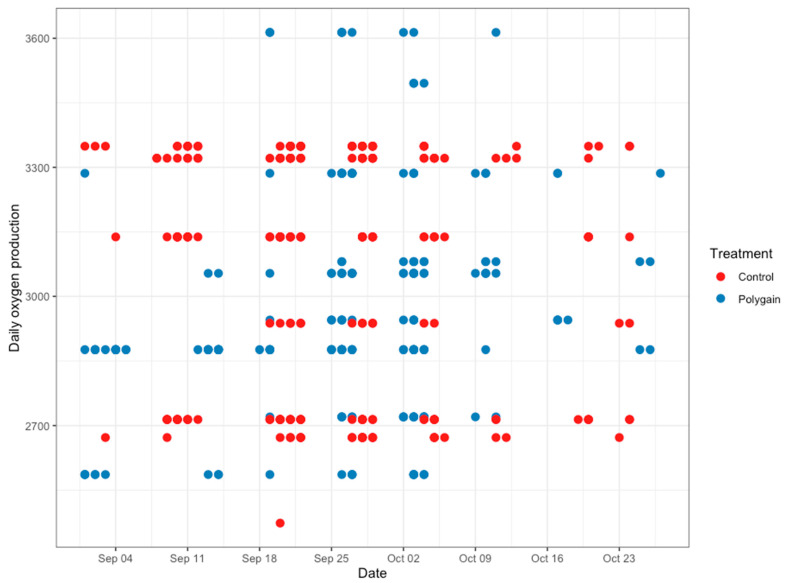
Average daily oxygen emission (g/calf/day) of control and Polygain™ supplemented calves during the study period.

**Figure 7:**
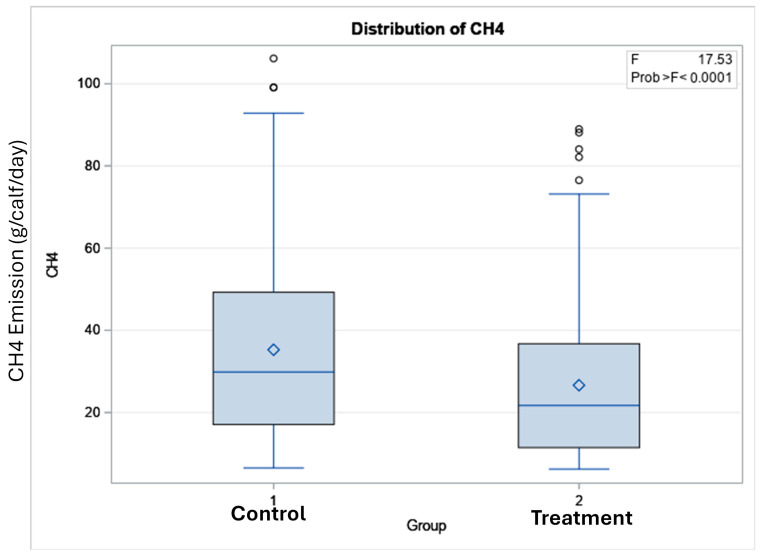
Box plot of methane emissions of experimental calves in the control (standard pellet) and treatment (Polygain™ supplemented pellet) groups.

The total number of visits to the GEM by each animal is shown in Figure 3. In terms of the potential benefits of feeding Polygain™ (PG) to growing calves, carbon dioxide (CO_2_) emission and oxygen (O_2_) emission were similar (*p* > 0.05) among the two groups (Table 2). The control animals had consistently a higher daily average methane production compared to PG-supplemented animals from the beginning to the end of the trial (Figure 4). The daily average CO_2_ and O_2_ emissions also showed similar and consistent variation among the control and treatment groups over the measurement period (Figure 5 and Figure 6). There was a significant (*p* < 0.001) effect of PG supplementation on the enteric CH_4_ emission in calves (Table 2), and the production of CH_4_ was lower in calves supplemented with the PG (26.66 ± 2.06 g/day) as compared to the control group (35.28 ± 1.39 g/day, *p* < 0.001; Figure 7). The CO_2_/O_2_ ratio in the treatment and control groups differed significantly (*p* < 0.001), being 235 ± 14 and 183 ± 9.6 in the treatment and the control group, respectively Table 2). Neither H_2_ nor H_2_S were exhaled in either group.

Pearson correlation coefficients between the three GHGs studied were positive and significant, with correlations between CH_4_ and CO_2_ being higher than those between CH_4_ and O_2_ (Table 3).

## 4. Discussion

The lack of variation in both the initial and final weight of control and treatment calves indicates similar growth performance in the two groups. The average daily gain of control calves was not statistically different than that of calves on the treatment diet; in fact, the difference was rather marginal (1.09 vs. 0.97 kg/calf/day). This seems to suggest that methane emissions do not always directly slow down growth but indicate inefficiencies in feed conversion. In practice, high methane production requires animals to consume more feed to achieve the same growth rate as low-emission animals, creating an indirect impact on growth efficiency. However, high-digestibility diets may mitigate some of this energy loss, allowing animals to sustain good growth rates despite higher methane emissions [35,36]. Anti-methanogenic feed supplements were tested in adult animals. However, rumen development occurs in calves at 6 months. Thus, in this study, we sought to identify the impact of feed supplements in reducing enteric methane production in early rumen development. Enteric CH_4_ emission results from microbial fermentation of feed components. The highly significant (*p* < 0.001) effect of Polygain™ (PG) supplementation on enteric methane emission, with lower production of CH_4_ in calves supplemented with PG compared to the control group, can be attributed to the anti-methanogenic properties of PG. In a previous study by our group, we reported that feeding Polygain™ to sheep reduced CH_4_ emissions without compromising intake or daily gain [23]. This is an important finding contributing to efforts aimed at reducing CH_4_ emission from ruminant livestock. As CH_4_ has 23 times the global warming potential of CO_2_ and a shorter atmospheric life (12 years for CH_4_ compared to 50–200 years for CO_2_), cutting CH_4_ emissions can reduce the impact of GHGs on global warming faster than focusing on CO_2_ alone [37].

Polyphenols, as plant-derived secondary metabolites, play a crucial role in modulating rumen fermentation and digestion efficiency in ruminants. For instance, polyphenols exhibit antimicrobial properties, selectively inhibiting certain rumen microbes, including proteolytic bacteria and methanogens. This can reduce populations of essential fibre-digesting bacteria like *Fibrobacter succinogenes* [38]. Condensed tannins also form complexes with proteins, reducing their degradation in the rumen and enhancing amino acid availability post-ruminally [39].

Our findings also highlight the need to preserve biological diversity, as various plant components are characterized by their methane reduction potential in animals. Ideal feed additives are those that can increase production, enhance net energy balance, and reduce methane emission [40,41]. Although plant extracts work well in reducing methane emissions, sustainability must also be considered, especially regarding sourcing and growing plants. The ability of plant components to reduce enteric CH_4_ emissions from ruminants depends on the amount of bioactive compounds in the plant, which in turn depends on its availability and sustainability, as well as the methods used to harvest, transport, store, and process it into feed ingredients [3].

Enteric methane (CH_4_) is produced by methanogens, a group of Archaea found in the rumen and hindgut of ruminant animals. Introducing feed additives to mitigate enteric methane from ruminants shows potential for reducing agricultural GHG emissions and improving ruminant productivity [40]. Supplements such as seaweed, *Asparagopsis*, legumes (*Desmanthus* or *Leucaena* species), brown algae *Ascophyllum nodosum*, essential oils (garlic and citrus extract), and 3-nitrooxypropanol (3-NOP), have demonstrated methane emissions reduction potential [5,40]. Feeding concentrate diets high in energy substrates (non-structural carbohydrates) reduces CH_4_ emission (g/d and g/kg DMI); whereas high-fibre diets (forages) result in increased CH_4_ emissions [1,42]. The fibre hydrolysis rate in the rumen, the rumen pH, and the feed particle size can all explain the dietary influence on enteric methane emissions [43]. The rate at which the carbohydrates in the concentrate and fibre ferment varies, and the latter produces lower pH values that partially limit methanogens [44].

Thus, dietary manipulation influences CH_4_ production by directly influencing the rumen microbiome, providing an opportunity to reduce CH_4_ emissions from cattle production systems. Increased animal productivity was shown to result from reduced enteric CH4 production per unit of production (milk and ADG) and improved feed efficiency [1]. Improved nutrition, management, reproduction, or genetics can reduce CH_4_ production per unit of meat or milk [45]. The nature and rate of carbohydrate fermentation influence the proportion of individual VFAs formed and thus the amount of CH_4_ produced. Fermentation of structural carbohydrates results in a greater CH_4_ loss than fermentation of soluble sugars and starches. Thus, mitigation of CH_4_ emissions can be effectively achieved by strategies that improve animal production efficiency, reduce feed fermentation per unit of product, or change the fermentation pattern in the rumen [42]. While management and dietary solutions to reduce enteric methane emissions have been extensively researched, animal breeding to exploit natural variations in methane emissions should offer a cost-effective, permanent, and cumulative solution [46]. Genetic selection aimed at reducing CH_4_ emissions from dairy cows promises to be a sustainable option [3,5,43,47], worth exploiting.

The significant positive correlation between CO_2_ and CH_4_ in this study aligns with previous findings, indicating that CO_2_ production data can accurately predict CH_4_ emissions, facilitating large-scale data generation for management and genetic evaluations in the dairy industry [48]. The CH_4_:CO_2_ ratio is particularly useful in identifying low CH_4_-producing cows [49,50]. In this study, we computed its inverse, the CO_2_/CH_4_ ratio in expired gases, as a potential index of energy metabolism in grazing animals [51,52,53]. Our findings suggest that feeding calves Polygain™ improves their energy metabolism and reduces methane production.

## 5. Conclusions

Polygain™ (PG) supplementation (10 g/calf/day) in calves reduced their average CH_4_ emission per day and did not substantially affect their growth or development. PG feeding resulted in a 24% reduction of CH_4_ production with little effect on the average daily gain of experimental calves (0.97 kg/day compared to 1.09 kg/day for control animals). Hence, PG can be considered a suitable feed additive to reduce CH_4_ emissions. This study further confirms the anti-methanogenic potential of PG and offers another dietary additive that can help the livestock industry achieve its CH_4_ emission reduction targets and promote sustainable production in the face of climate change. By introducing CH_4_-mitigating feed additives in calves, the composition and activity of the rumen microbial community may be influenced, resulting in reduced methane production. This early intervention needs to be investigated further to develop a potential long-term methane mitigation strategy. Future studies should also consider increasing the number of experimental calves, adding different levels of Polygain™ (10 g, 15 g, and 20 g/calf/day for instance) to the diet of calves and evaluating the potential benefits of feeding Polygain™ to growing calves in terms of their growth and blood antioxidant profiles. Additionally, our research group hopes to explore other potential strategies such as genetic selection and breeding for low methane-emitting dairy cattle as a long-term sustainable strategy for climate-smart dairy cattle production.

## Figures and Tables

**Table 1:** Average calf body weight ± SD * (n) of experimental calves by group.

Parameter	Control Group	Treatment Group
Initial body weight (kg)	185.3 ± 25.5 (12)	192.0 ± 27.2 (12)
Final body weight (kg)	251.0 ± 31.6 (12)	250.2 ± 25.9 (12)
Average daily gain (kg/d)	1.09 ± 0.2 (12)	0.97 ± 0.1 (12)

* SD = standard deviation; n = sample size.

**Table 2:** Mean concentration of different gases in the exhaled air in g/day/animal (±SE).

Parameter	Control (g/Day)	n	Treatment (g/Day)	n	*p* Value
CH_4_	35.3 ^a^ ± 22.4	219	26.7 ^b^ ± 18.1	183	<0.001
CO_2_	4125.0 ± 765.2	219	4164.2 ± 768.2	183	0.61
O_2_	3044.2 ± 535.5	219	2973.0 ± 563.8	183	0.19
CO_2_/CH_4_	183.1 ^b^ ± 138.9	219	235.3 ^a^ ± 146.7	183	<0.001
CO_2_/O_2_	1.4 ^a^ ± 0.01	219	1.3 ^b^ ± 0.01	183	<0.001
O_2_/CH_4_	135.4 ^b^ ± 102.8	219	168.6 ^a^ ± 153.2	183	0.002

The values are regression estimates reported with standard errors. SE = standard error; n = sample size. Within rows, means bearing different superscripts are significantly different (*p* ≤ 0.05).

**Table 3:** Pearson correlation coefficients between GHGs and their ratios.

	CO_2_	CH_4_	O_2_	CO_2_:CH_4_	CO_2_:O_2_	O_2_:CH_4_
CO_2_	1	0.2578	0.8697	0.0873	0.2382	0.0535
CH_4_	<0.0001	1	0.2209	−0.7744	0.0599	−0.7748
O_2_	<0.0001	<0.0001	1	0.0615	−0.2597	0.0992
CO_2_: CH_4_	0.0848	<0.0001	0.2186	1	0.0592	0.9861
CO_2_: O_2_	<0.0001	0.2305	<0.0001	0.2362	1	0.0801
O_2_: CH_4_	0.0535	<0.0001	0.0468	<0.0001	0.1087	1

Pearson correlation coefficients are indicated in the upper diagonal, with *p*-values in the lower diagonal.

## Data Availability

The data presented in this study are available on request from the corresponding author. The data are not publicly available due to institutional restrictions.
